# Medical Items

**Published:** 1911-11

**Authors:** 


### MEDICAL ITEMS.

### IN NERVOUS EXCITEMENT.

The principal indications for Peacock's Bromides are, of course, Epilepsy, Uterine Congestion, Headache and all Neuroses. Being a safe and certain nerve sedative, it will be found of most valuable aid whenever the mental functions are overtaxed, producing insomnia. Peacock's Bromides do not compel sleep, like hypnotics, but by allaying the existing nervous excitement, whether due to mental strain, worry or anxiety, it promotes sleep in a normal manner. Unlike the effects from hypnotics, the patient awakens refreshed, with a clear head, and does not suffer from unpleasant sequelae the following day. The overstimulation of the cerebral functions from alcohol yields promptly to the soothing action of this preparation.

### BROMISM.

When Peacock's Bromides are given over a prolonged period, as is often necessary in epilepsy and nervous diseases, its advantages over commercial substitutes are unmistakable. While bromism cannot be absolutely prevented in patients havng an idiosyncrasy toward bromides, it has been positively demonstrated that Peacock's Bromides can be given with greater freedom from untoward action, and that frequently the preparation can be employed to continue bromide treatment after the commercial bromides were necessarily discontinued. This severe trial is perhaps the most convincing evidence of its superiority.

### AN IDEAL PURGATIVE MINUS CATHARTIC INIQUITIES.

This elegant product, Prunoids, represents a real advance in the therapy of intestinal constipation. No one can use this remedy without being impressed with its prompt effects, convenience of use and surprising absence of undesirable consequences. The tablets are edible, pleasant to take, and will always be found to be a safe and positive eliminating agent in either toxic or non-toxic conditions of the intestines. They do not excessively excite peristalsis and therefore do not produce griping or irritation of the gastro-intestinal mucous membrane. It is certainly a scientific achievement in the successful treatment of constipation, for "after-constipation" will not result from its use, and in the language of Dr. J. P. Hawes, it proves to be "a laxative that is pleasant to take, does its work nicely, and QUITS there."

### A PERSUASIVE TONIC—NOT A THERAPEUTIC LASH.

Clinical observations demonstrate Cactina Pillets to be a mild tonic stimulant to the heart, acting both on the mechanism and directly upon the heart muscle. Its continued use will promote cardiac nutrition and overcome atony of the heart muscle. And in this assistance it affords the heart and circulation there is absolutely no danger of creating untoward symptoms or annoyance to the patient.

